# Long-term exposure to microgravity impairs vestibulo-cardiovascular reflex

**DOI:** 10.1038/srep33405

**Published:** 2016-09-16

**Authors:** Hironobu Morita, Chikara Abe, Kunihiko Tanaka

**Affiliations:** 1 Department of Physiology, Gifu University Graduate School of Medicine, Gifu 501-1194, Japan; 2 Department of Radiotechnology, Gifu University of Medical Science, Gifu 501-3892, Japan

## Abstract

The vestibular system is known to have an important role in controlling blood pressure upon posture transition (vestibulo-cardiovascular reflex, VCR). However, under a different gravitational environment, the sensitivity of the vestibular system may be altered. Thus, the VCR may become less sensitive after spaceflight because of orthostatic intolerance potentially induced by long-term exposure to microgravity. To test this hypothesis in humans, we investigated the ability of the VCR to maintain blood pressure upon head-up tilt before and after a 4–6 months stay on the International Space Station. To detect the functional state of the VCR, galvanic vestibular stimulation (GVS) was applied. As GVS transiently interrupts the vestibular-mediated pressor response, impaired VCR is detected when the head-up tilt-induced blood pressure response does not depend on GVS. During the first 20 s of head-up tilt, a transient blood pressure increase (11.9 ± 1.6 mmHg) was observed at pre-spaceflight but not at 1–4 days after return from spaceflight. The magnitude of VCR recovered to the pre-spaceflight levels within 2 months after return. These results indicate that long-term exposure to microgravity induces VCR impairment, which may be involved in a mechanism of spaceflight-induced orthostatic intolerance.

The vestibular otolith organs detect the sum of linear accelerations and help regulate a variety of biological functions such as ocular movement (vestibulo-ocular reflex)^1^,^2^, body stability (vestibulo-spinal reflex)^3^,^4^, and sympathetic nerve activity (vestibulo-sympathetic reflex). This type of sensing is also involved in the regulation of arterial blood pressure (BP) (vestibulo-cardiovascular reflex, VCR)^5^,^6^,^7^, as well as that of bone and muscle metabolism^8^,^9^,^10^. However, the vestibular system is known to be highly plastic, i.e., its sensitivity might be altered if the subject is exposed to a different gravitational environment. Understanding the plasticity of the vestibular system and its effect on vestibular-mediated functions is essential in the study of microgravity-induced medical complications such as body instability, orthostatic hypotension, muscle atrophy, and bone loss; these conditions are major complications associated with spaceflight^11^,^12^,^13^,^14^,^15^,^16^ and should be addressed before assigning astronauts on space missions that require traveling longer distances or spending longer periods of time under microgravity conditions.

While there is a considerable body of evidence regarding the microgravity-induced plastic alteration of the vestibulo-ocular and vestibulo-spinal reflexes^1^,^2^,^3^,^4^, the effect of such plastic alteration on the VCR has not been assessed to date. Recently, Hallgren and colleagues^17^ found a significant correlation between decreased otolith function and reduced BP response upon head-up tilt (HUT) on return from spaceflight. They suggested that a deconditioned otolith system causes orthostatic intolerance; however, they did not directly evaluate the sensitivity or magnitude of the altered VCR. We hypothesized that long-term exposure to microgravity induces plastic alteration of the VCR, and that vestibular-mediated pressor response is impaired after spaceflight.

In animal experiments, the magnitude of VCR had been evaluated by comparing BP responses to gravitational changes between animals with intact vestibular system and animals with vestibular lesion. However, invasive methods such as the vestibular lesion cannot be used in human studies. Thus, an alternative, less invasive method is required to study the magnitude of VCR in humans. For this purpose, we used galvanic vestibular stimulation (GVS), which is electrical stimulation applied externally to the primary vestibular afferents^18^. Continuously-applied GVS obscures the vestibular input associated with gravitational change, and then transiently interrupts the vestibular-mediated pressor response^19^,^20^. Therefore, it is possible to evaluate the magnitude of the VCR-mediated pressor response by comparing the HUT-induced BP response with and without GVS. If the VCR operates, BP response to HUT without GVS is expected to be higher than that to HUT with GVS; on the other hand, if the VCR does not operate, the BP response to HUT is expected to be independent of the application of GVS.

Therefore, the purpose of the present study was to examine the impact of long-term exposure to microgravity on the plasticity of the vestibular system with respect to the VCR-mediated pressor response. To perform this investigation in humans, we measured the HUT-induced BP response with and without application of GVS.

## Results

Six male astronauts, aged 38–53 years, were recruited for this study. The subjects spent 156 ± 9 days (range, 127–188 days) on the International Space Station (ISS). Data were collected on four different occasions for each subject: 2–4 months before launch (P0); 1–4 days after return (P1); 11–15 days after return (P2); and 2 months ± 12 days after return (P3).

BP and heart rate at supine rest (no HUT) tended to increase after the stay on the ISS (Table 1); however, the differences did not reach a statistical significant level for BP (systolic BP, *F*_3,15_ = 2.079, *P* = 0.146; diastolic BP, *F*_3,15_ = 1.786, *P* = 0.193; mean BP, *F*_3,15_ = 2.459, *P* = 0.103) or for heart rate (*F*_3,15_ = 2.150, *P* = 0.137).

### Blood pressure response to HUT

**Figure 1:**
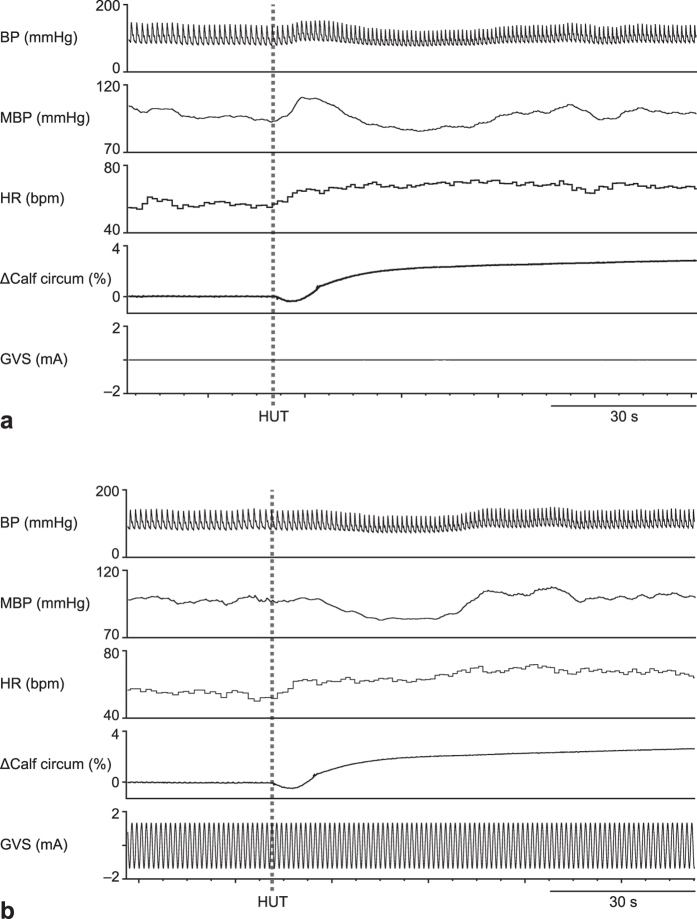
Typical baseline (pre-flight) responses of arterial blood pressure (BP), mean BP (MBP), heart rate (HR), Δcalf circumference, and amplitude of galvanic vestibular stimulation (GVS) to the head-up tilt (HUT) test, either without GVS (**a**) or with strong GVS (**b**). HUT was started at the broken line and reached 60° at 6 s after the start.

**Figure 2:**
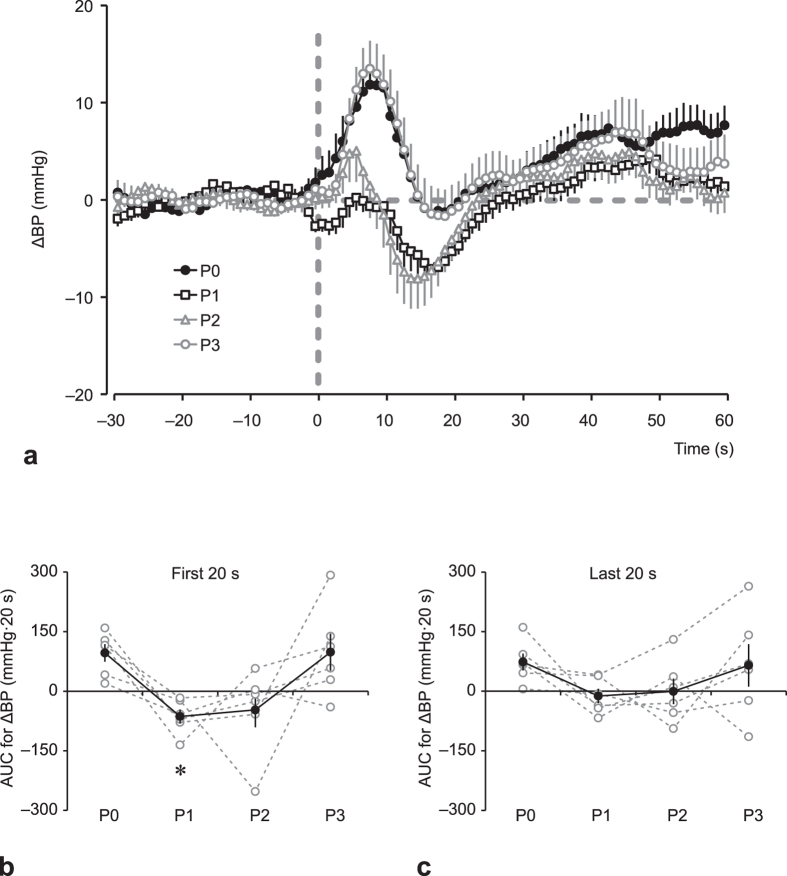
Averaged arterial blood pressure responses (ΔBP) to head-up tilt (HUT) test at: 2–4 months before launch (P0, closed circle); 1–4 days after return (P1, open square); 11–15 days after return (P2, open triangle); and 2 months ± 12 days after return (P3, open circle). (**a**) Each data point is presented as a difference from the 30 s average just prior to the induction of HUT. Area under the curve (AUC) of ΔBP during the first 20 s of HUT (**b**) and the last 20 s of HUT (**c**). Open circle and dashed line represent individual data, and closed circle and solid line represent mean ± standard error of the mean for 6 subjects. *P < 0.05 vs. P0.

HUT experiments were performed under three different conditions: without GVS; with weak GVS; and with strong GVS. Figure 1a shows the response of BP to HUT without GVS at P0 (2–4 months before launch). From the onset of HUT, BP transiently increased; such transient increase in BP was not observed for HUT under strong GVS (Fig. 1b). Overall data regarding change in BP (ΔBP) without GVS before and after the ISS stay are shown in Fig. 2a. The transient increase in BP (+11.9 ± 1.6 mmHg) from the onset of HUT seen at P0 was not observed at P1 (1–4 days after return), when BP decreased by 7.1 ± 1.9 mmHg at 17 s after the onset of the HUT, and recovered to pre-HUT levels at 25–30 s after the start of the HUT. A small transient increase in BP was seen at P2 (11–15 days after return), after which BP decreased below the pre-HUT levels, and then recovered. The BP response at P3 (2 months ± 12 days after return) was comparable with that at P0. The area under the curve (AUC) for ΔBP during the first 20 s was significantly affected by the ISS stay (Fig. 2b; *F*_3,15_ = 6.031, *P* = 0.0066). A post-hoc analysis, comparing the AUC for ΔBP at P0 with those at P1, P2, and P3 revealed a significant decrease in AUC for ΔBP at P1. At the end of the 2-min HUT, BP settled at a level slightly higher than the pre-HUT level (pre-HUT, 94.6 ± 4.4 mmHg; last 20 s of the 2-min HUT, 99.1 ± 5.0 mmHg; *P* = 0.01387, paired t-test). The AUC for ΔBP during the last 20 s of the 2-min HUT tended to decrease after the ISS stay, but such difference was not statistically significant (Fig. 2c; *F*_3,15_ = 3.002, *P* = 0.0535).

### Effects of strong GVS

**Figure 3:**
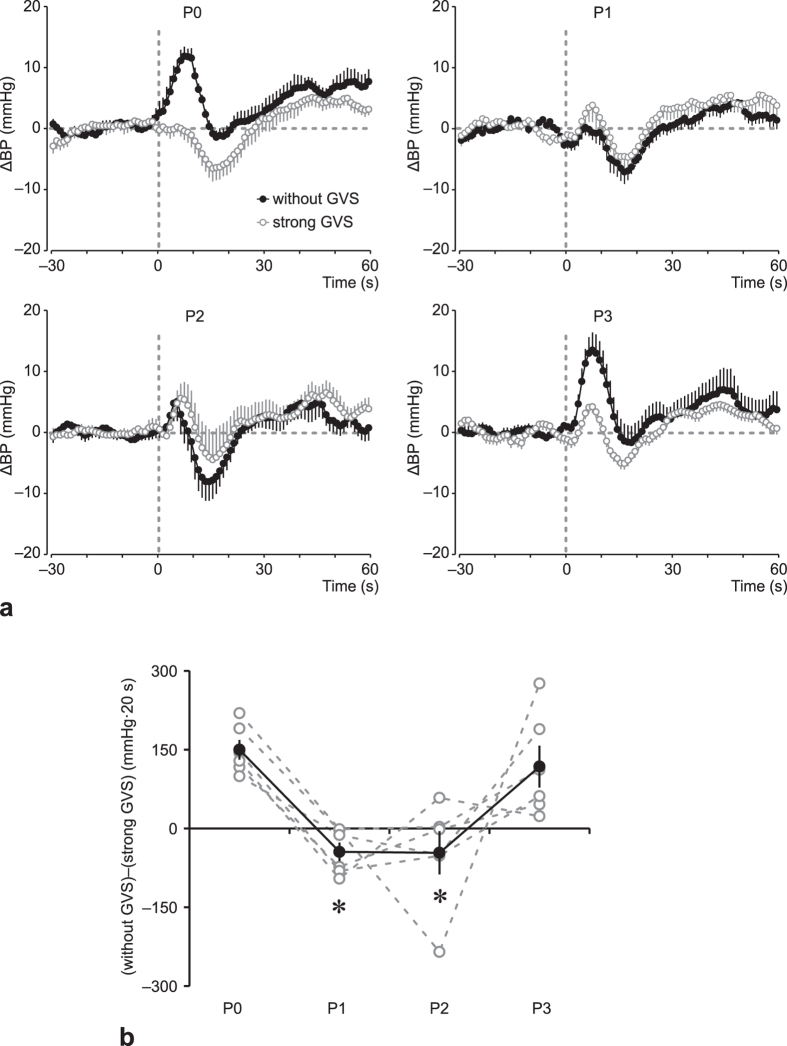
Averaged arterial blood pressure responses (ΔBP) to head-up tilt (HUT) test without GVS (closed circle) and with strong GVS (open circle) at 2–4 months before launch (P0); 1–4 days after return (P1); 11–15 days after return (P2); and 2 months ± 12 days after return (P3) (**a**). Each data point is presented as a difference from the 30 s average just prior to the induction of HUT. Sum of differences in ΔBP between the initial response (within the first 20 s) to HUT without GVS and HUT with strong GVS [(without GVS)−(strong GVS)] (**b**). Open circle and dashed line represent individual data, and closed circle and solid line represent mean ± standard error of the mean for 6 subjects. *P < 0.05 vs. P0.

Figure 3a shows ΔBP for HUT under strong GVS and without GVS. Figure 3b shows the differences in BP over the first 20 s due to HUT under strong GVS and without GVS from P0 to P3, reflecting a positive difference in VCR across the measurements. However, this difference was not seen at P1 and P2, suggesting that VCR was inactive upon HUT at P1 and P2, but recovered its activity at P3. The sum of differences of ΔBP between HUT without GVS and under strong GVS during the first 20 s after HUT was significantly affected by the ISS stay (*F*_3,15_ = 9.294, *P* = 0.0010). A post-hoc analysis revealed a significant decrease in such difference at P1 and at P2, but not at P3.

### Effects of weak GVS

**Figure 4:**
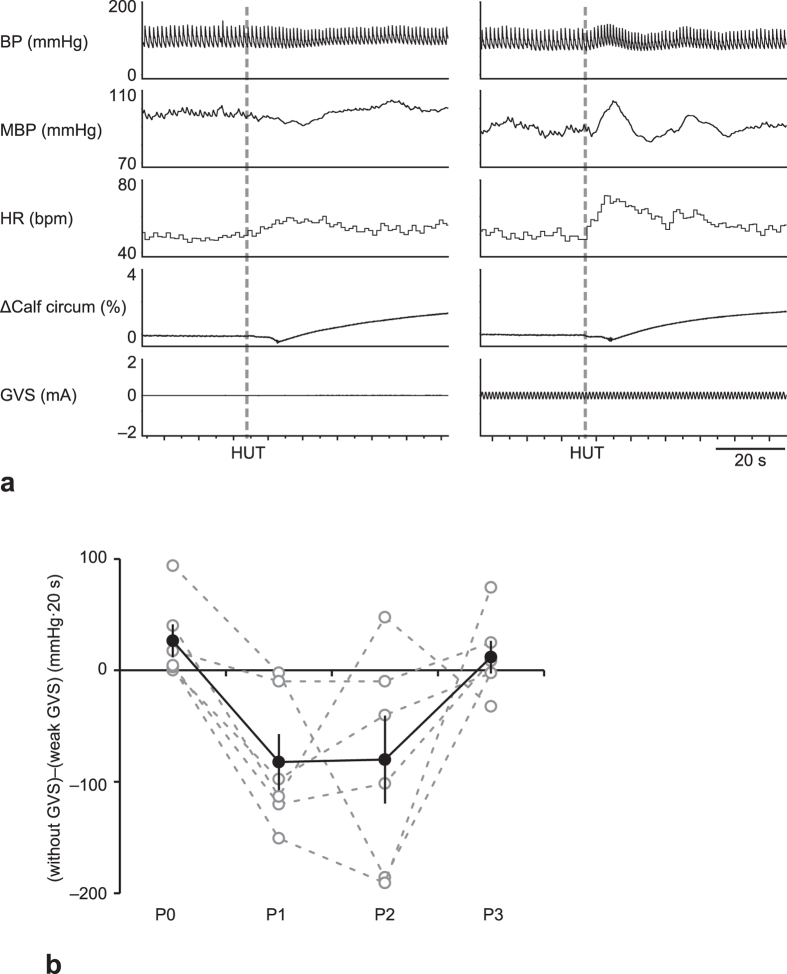
Typical responses of arterial blood pressure (BP), mean BP (MBP), heart rate (HR), Δcalf circumference, and amplitude of galvanic vestibular stimulation (GVS) to the head-up tilt (HUT) test, under without GVS (a, left panel) and with weak GVS ((**a**), right panel) at 1–4 days after return (P1). Sum of differences in ΔBP between the initial response (within 20 s) to HUT without GVS and with weak GVS [(without GVS)−(weak GVS)] (**b**). Open circle and dashed line represent individual data, and closed circle and solid line represent mean ± standard error of the mean for 6 subjects.

Typical responses of BP to HUT with or without weak GVS at P1 are shown in Fig. 4a. The transient increase in BP was not seen in HUT without GVS; rather, BP decreased at the onset of HUT. On the other hand, BP transiently increased when weak GVS was applied. The sum of differences in ΔBP between HUT without GVS and under weak GVS during the first 20 s of HUT are shown in Fig. 4b. At P0 and P3, such differences were close to zero (26.5 ± 14.8 and 11.8 ± 14.7 mmHg·20 s, respectively), indicating that BP responses to HUT under weak GVS or without GVS were very similar. However, those differences were negative at P1 and P2, indicating that BP response for HUT under weak GVS was higher than that for HUT without GVS. In particular, those differences were considerably high for 4 of 6 astronauts, with values from −82 to −151 mmHg·20 s; for the other two astronauts the differences were −2 and −10 mmHg·20 s. Thus, such difference was significantly affected by the ISS stay (*F*_3,15_ = 4.677, *P* = 0.0169); however, post-hoc analysis could not detect differences between values at P0 and values at P1, P2, or P3.

### Baroreflex sensitivity

**Figure 5:**
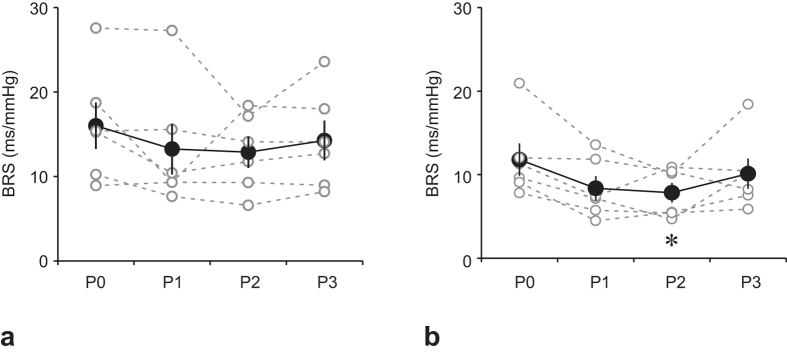
Changes in baroreceptor-heart rate reflex sensitivity (BRS) at supine rest (**a**) and during HUT (**b**). Open circle and dashed line represent individual data, and closed circle and solid line represent mean ± standard error of the mean for 6 subjects. *P < 0.05 vs. P0.

Figure 5a shows changes in baroreceptor-heart rate reflex sensitivity (BRS) at supine rest, and Fig. 5b shows changes in BRS during HUT. BRS at rest tended to decrease after the ISS stay; however, this difference did not reach the statistical significance level (*F*_3,15_ = 1.487, *P* = 0.2584). On the other hand, BRS during HUT was significantly affected by the ISS stay (*F*_3,15_ = 4.401, *P* = 0.0207). A post-hoc analysis revealed a significant decrease in BRS at P2 but not at P1 or P3.

## Discussion

The present study is the first to investigate the impact of long-term exposure to microgravity on the human VCR and its re-adaptation to the Earth’s gravitational field. The most significant finding is that the VCR was completely absent on return from spaceflight, but it recovered gradually within 2 months.

The vestibular system is known to play a significant role in regulating BP during postural changes, which imply changes in the amount and direction of gravity^21^. In animal experiments, hypergravity- or microgravity-induced BP responses were abolished by vestibular lesion, and these animals developed orthostatic intolerance^5^,^19^,^22^,^23^. To examine the significance of VCR in humans, various stimuli can be applied to the vestibular system while measuring the sympathetic nerve activity in the muscles. The results of such studies involving GVS, head-down rotation, off-vertical axis rotation, and horizontal linear acceleration indicated that the vestibular system modulates sympathetic nerve activity in humans^24^,^25^,^26^,^27^,^28^,^29^. Furthermore, it was shown that patients who experienced dizziness and had an abnormal deviation of the subjective visual vertical (which is an indicator of imbalance of the otolith function) exhibited a greater decrease in systolic BP upon standing compared to that exhibited by healthy control subjects or even patients who experienced dizziness but had a normal deviation of the subjective visual vertical^30^. Therefore, this vestibular-mediated sympathetic response might have a significant role in regulating BP upon standing.

In the present study, the magnitude of VCR was evaluated by comparing BP response to HUT with or without strong GVS. In our subjects, strong GVS evoked robust vestibular illusion of swinging from side to side. In rats, application of strong GVS reduced the microgravity- or hypergravity-induced BP response, and the effect was comparable to that of vestibular lesion; on the other hand, application of strong GVS had no influence on the air jet-induced pressor response^19^. In humans, strong GVS impaired the vestibular-mediated pressor response (BP response to HUT), but it did not affect the BP response to lower-body negative pressure, which induced a similar degree of footward fluid shift without alteration of the vestibular input^20^. In the present study, HUT conducted a few months before launch induced a transient increase in BP (ΔBP at P0), which was abolished or significantly decreased upon application of strong GVS. The difference between ΔBP values obtained with and without application of strong GVS may represent the vestibular-mediated BP response. Within days after return (ΔBP at P1 and P2), HUT induced small and transient fall in BP, suggesting that long-term stay in the ISS resulted in significantly impaired or completely absent VCR.

The otolith organs sense head tilt and send information to the brain about spatial orientation. Input to the otolith organs might be minimal during exposure to microgravity, forcing the otolith organs to adapt and enable spatial orientation under microgravity conditions. When the astronauts re-enter the Earth’s gravitational field, the otolith-mediated reflex might be altered. Hallgren and colleagues recently investigated the effect of 6 months of exposure to microgravity on the otolith-mediated ocular response in a considerably large group of astronauts (n = 25), and found that ocular counter-rolling response was decreased at 2–5 days after return, but it recovered to preflight levels at 9 days after return, indicating that the peripheral otolith system can achieve full recovery within 9 days^2^. However, in the present study, VCR had not recovered within 11–15 days after return (at P2). A possible explanation for this discrepancy could be the existence of a compensatory mechanism with respect to ocular counter-rolling response. Since gain of vestibular-mediated eye movement is augmented by somatosensory inputs or adequate training^31^,^32^, the rehabilitation process after the return might have hastened the re-adaptation to the Earth’s gravitational field.

Both BRS at supine rest and HUT-induced BRS tended to decrease after return from spaceflight, but only the change in HUT-induced BRS reached statistical significance. Interaction between the VCR (and/or the vestibulo-sympathetic reflex) and the baroreflex has been reported in animals and humans^7^,^23^,^33^. In humans, head-down rotation-induced sympathetic nerve activity in the muscles is greater upon HUT than in prone position, and also greater when cardiopulmonary and arterial baroreceptors are unloaded by lower body negative pressure^7^,^33^. We previously conducted an open-loop baroreflex analysis on anesthetized rats with intact or lesioned vestibular organs; the rats were placed in the prone or HUT position, and the results demonstrated that the vestibular system elicits sympathoexcitation, shifting the baroreflex neural arc to a higher sympathetic nerve activity, and maintaining BP during HUT^23^. Thus, if the VCR does not operate during HUT, the maximal gain of baroreflex might be lower, which might contribute to the significantly reduced BRS observed after return during HUT, but not at supine rest.

We used weak GVS as a negative control for strong GVS because the subjects experienced head sway during strong GVS, but no noticeable alteration during weak GVS. We thus assumed that, prior to space flight, weak GVS had no effect on HUT-induced BP response, whereas strong GVS suppressed HUT-induced BP response. However, BP responses with weak GVS at P1 and P2 were ameliorated in 4 of 6 astronauts included in our study, which was an unexpected result. In this regard, it is reasonable to consider stochastic resonance, which is a phenomenon wherein the response of a nonlinear system to a weak periodic input signal is optimized by adding imperceptible amounts of noise to the system^34^. In particular, it was shown that an imperceptible level of white noise GVS is effective in improving postural stability and gait performance in patients with bilateral vestibular dysfunction^35^,^36^. In the present study, we used a sinusoidal (1.0 Hz) current with an amplitude of 80% of the perception threshold as weak GVS; the stimulation pattern and amplitude were not optimized for stochastic resonance. Nevertheless weak GVS improved HUT-induced BP response after return in some subjects, which suggests that optimized white noise GVS might serve as a new countermeasure for orthostatic intolerance or body instability after return from spaceflight.

The vestibular otolith organs detect postural (gravity) changes and reflexively increase sympathetic nerve activity with a brief latency^27^, and then induce an increase in BP to counteract the subsequent BP decrease due to footward fluid shift. Thus, the VCR acts as a feedforward BP regulator against gravitational change. In other words, the VCR regulates BP based on gravitational change, not based on BP change; this gives rise to control errors, which are detected and corrected by the negative feedback system (baroreflex)^5^. Accordingly, to evaluate the VCR uncorrected by the baroreflex, we examined early HUT-induced BP response (1–20 s) for a total HUT duration of 2 min. This procedure did not allow us to evaluate orthostatic intolerance, which, in astronauts, is typically evaluated by a 10-min stand test^11^,^16^. Thus, we could not conclude whether the impaired VCR contributes to orthostatic intolerance, which represents a limitation of our study. The underlying mechanisms involved in post-spaceflight orthostatic intolerance are multi-factorial; several aspects have been suggested to be key factors affecting orthostatic intolerance, including hypovolemia, loss of circulating fluid via the capillary, alterations in baroreflex sensitivity, less vasoconstrictor response, cardiac muscle atrophy, changes in peripheral adrenergic receptor responses, and plastic alteration of the VCR^11^,^37^,^38^,^39^,^40^,^41^,^42^. In the present study, we demonstrated that long-term exposure to microgravity induces an impairment in VCR at return to the Earth, which might contribute, at least in part, to post-spaceflight orthostatic intolerance, since HUT-induced BP response was lower at P1 and P2, in which the VCR did not operate.

We found that the VCR may be completely abolished by long-term exposure to microgravity conditions, but it recovers gradually within 2 months after return. Weak GVS can serve as a new countermeasure for orthostatic intolerance and body instability after return from spaceflight.

## Methods

### Subjects

All subjects gave their written consent to undergo the experimental procedures part of the present study after being informed of their aim and nature, and informed consent was obtained from all subjects. The study was conducted under the guidelines issued by the Committee for the Protection of Human Subjects at the Johnson Space Center of the National Aeronautics and Space Administration (NASA). The study received approval from the Japan Aerospace Exploration Agency (JAXA) Research and Medical Committees, as well as from the Ethics Committee of the Gifu University Graduate School of Medicine.

Data were collected at NASA’s Johnson Space Center in Houston, Texas. As astronauts typically participate in several research investigations simultaneously, we wished to ensure that there would be no interference between our study and other similar investigations potentially taking place during the same period. Therefore, the subjects were instructed to abstain from taking vasoactive drugs for one hour, from heavy meals for 4 hours, and from vigorous exercise for 12 hours prior to data collection. Moreover, at the end of each experiment, we asked the astronauts to report all drugs they had received within the 6 hours prior to testing; we noted that no medicines had been administered.

### Head-up tilt experiment

Each subject lay supine on a tilt table, with the body supported by a saddle in order to prevent leg muscle contraction and caudal movement. All measurements were performed noninvasively using electrodes or probes attached to the surface of the skin.

Beat-to-beat BP was recorded via finger photoplethysmography (Finometer, Finapres Medical Systems, Amsterdam, The Netherlands). Finger BP was calibrated using the brachial BP, and height-corrected for the difference between the hydrostatic pressure of the heart and that of the finger. Calf circumference was measured using a mercury strain gauge (EC6, Hokanson, Bellevue, WA, USA). A pair of surface electrodes for GVS was placed on the bilateral mastoid processes, and the subject’s eyes were covered by an eye mask. Next, a sinusoidal current (1.0 Hz) was applied in order to induce GVS. The stimulating amplitude for weak GVS was set to 80% of the perceptible threshold (0.3–0.5 mA), while the stimulating amplitude for strong GVS was set between the perceptive threshold and the discomfort threshold (4–5 mA). We used weak GVS as a negative control for strong GVS because the subjects experienced head sway during strong GVS, but no noticeable alteration during weak GVS. All signals were recorded using an analog-to-digital converter (PowerLab, AD Instruments, New South Wales, Australia) set to a frequency of 1,000 Hz.

The HUT experiment consisted of three alternated measurement periods: a 1-minute control period (tilt angle of 0°); a 2-minute HUT period (tilt angle of 60°); and a 1-minute recovery period (tilt angle of 0°). HUT experiments were performed under three different conditions: without GVS; with weak GVS; and with strong GVS. For each condition, 3–4 HUT repeated experiments were performed on each subject, and their BP responses were averaged. An interval between the repeated HUT experiments was given, lasting until the BP of the subject returned to the pre-HUT levels.

### Baroreflex sensitivity

BRS was calculated using a sequence method, for both spontaneous increases and spontaneous decreases in BP, at supine rest and during HUT^43^. The software (LabChart 7, AD Instruments, New South Wales, Australia) automatically extracted the systolic BP and pulse interval from the raw BP recording. Sequences with increased systolic BP associated with increased pulse interval, and those with decreased systolic BP associated with decreased pulse interval were analyzed. For each subject, regression slopes were determined for each beat sequence, and the mean value was computed. Finally, sequences were excluded in case they did not meet the threshold criteria of: changes in systolic BP of at least 1 mmHg per beat; concordant change in pulse interval of at least 6 ms per beat; having at least 3 beats in length; and exhibiting a correlation coefficient >0.9 (LabView 8.2, National Instruments, Austin, TX, USA).

### Statistical analysis

BP was averaged for every 1 s, and ΔBP for each subject was calculated as a difference between each 1-s data point and the 30 s average taken just before the subject underwent HUT. AUC was calculated as a sum of 20 data points of ΔBP: from 1 s to 20 s after the HUT, or from 101 to 120 s after the HUT. From 1 s to 20 s, the sum of the differences between each ΔBP of the HUTs without GVS and under strong GVS, as well as between each ΔBP of HUTs without GVS and under weak GVS were calculated. All data were presented as mean ± standard error of the mean (SEM). Repeated measures one-way ANOVA was applied. If the *F*-ratio indicated statistical significance, the Tukey-Kramer post-hoc test was used. For post-hoc test, the significance threshold was set at *P* < 0.05.

## Additional Information

**How to cite this article**: Morita, H. *et al.* Long-term exposure to microgravity impairs vestibulo-cardiovascular reflex. *Sci. Rep.* **6**, 33405; doi: 10.1038/srep33405 (2016).

## Figures and Tables

**Table 1:** Characteristics of the astronauts (n = 6), measured on four different occasions: 2–4 months before launch (P0); 1–4 days after return (P1); 11–15 days after return (P2); and 2 months ± 12 days after return (P3).

	P0	P1	P2	P3
Height (cm)	175.8 ± 3.1	175.5 ± 3.5	175.5 ± 3.5	175.7 ± 3.2
Body mass (kg)	78.5 ± 4.6	79.2 ± 4.7	79.4 ± 4.9	78.7 ± 4.8
Systolic BP (mmHg)	120.7 ± 2.7	129.3 ± 5.3	131.5 ± 4.5	121.4 ± 5.6
Diastolic BP (mmHg)	73.6 ± 3.5	78.9 ± 3.9	76.6 ± 3.4	69.9 ± 3.3
Mean BP (mmHg)	90.2 ± 3.1	98.0 ± 4.6	96.3 ± 3.9	87.9 ± 3.3
Heart rate (bpm)	55.3 ± 2.8	62.5 ± 4.0	59.9 ± 2.4	56.7 ± 2.4

Values are given as mean ± standard error of the mean. BP = blood pressure.
